# Dose intraarticular steroid injection increase the rate of infection in subsequent arthroplasty: grading the evidence through a meta-analysis

**DOI:** 10.1186/s13018-014-0107-2

**Published:** 2014-11-13

**Authors:** Dan Xing, Yang Yang, Xinlong Ma, Jianxiong Ma, Baoyi Ma, Yang Chen

**Affiliations:** Department of Orthopaedics Institute, Tianjin Hospital, 406 Jiefang Nan Street, Hexi District, Tianjin, 300211 China

**Keywords:** Steroid, Injection, Infection, Arthroplasty, Meta-analysis

## Abstract

**Background:**

Intraarticular steroid injections are widely used in joint arthritis. However, the data regarding an association between an increased risk for arthroplasty infection after an intraarticular steroid injection are still conflicting. We conducted a meta-analysis to evaluate the evidence from relevant studies that examine the relation between intraarticular steroid injections and infection rates in subsequent joint arthroplasty and to develop GRADE based recommendations for using the steroid before arthroplasty.

**Methods:**

A systematic search of all studies published through August 2014 was conducted using the MEDLINE, EMBASE, OVID, ScienceDirect and Cochrane CENTRAL databases. The relevant studies that examined the relation between intraarticular steroid injections and infection rates in subsequent joint arthroplasty were identified. Demographic characteristics, infection rates and clinical outcomes were manually extracted from all of the selected studies. The evidence quality levels and recommendations were assessed using the GRADE system.

**Results:**

Eight studies looking at hip and knee arthroplasties were included. Meta-analysis showed that patients with steroid injection before arthroplasty had a higher deep infection rate than patients without steroid injection (OR = 2.13, 95% CI 1.02-4.45), but no significant effect on superficial infection rate (OR = 1.75, 95% CI 0.74-4.16). The overall GRADE system evidence quality was very low, which lowers our confidence in their recommendations.

**Conclusions:**

Intraarticular steroid injections may lead to increased deep infection rates of subsequent joint arthroplasty but not the superficial infection rates. Due to the poor quality of the evidence currently available, further studies are still required.

## Introduction

Intraarticular steroid injections can be administered for diagnostic [1-4] and therapeutic reasons [5-8]. Particularly, they were widely used to alleviate inflammatory symptoms that can be associated with moderate or end-stage osteoarthritis of the joint. Compared with the injection of a long-acting anesthetic, steroids can be used for diagnostic purposes to distinguish intrinsic from extrinsic sources of pain such as that originating in the spine [1-4]. The duration and efficacy of pain relief then can be indicative of the source of pain [5-8]. Intraarticular steroid injections can be helpful in clinical practice when patients with moderate or end-stage osteoarthritis are not willing or suitable to undergo an arthroplasty in the short term [9].

However, there have been several adverse effects reported with the use of steroid, such as tendon rupture [10,11] and increased risk of joint infection in subsequent arthroplasty [12]. Given the potentially devastating outcomes of arthroplasty infection, determining whether such a relation exists is of high clinical importance. Several clinical studies have been conducted to examine this issue previously. Two studies [13,14] showed more infections in a group of patients who had an intraarticular steroid injection in the hip before they had an arthroplasty. While, other two studies [15,16] demonstrated that there was no increase in infection rates in patients who had arthroplasty after intraarticular injections of a steroid into the joint.

At present, the data regarding an association between an increased risk for arthroplasty infection after an intraarticular steroid injection are still conflicting. Therefore, the purpose of the present meta-analysis is to evaluate the evidence from relevant studies that examine the relation between intraarticular steroid injections and infection rates in subsequent joint arthroplasty and to develop GRADE (Grading of Recommendations, Assessment, Development, and Evaluation) based recommendations for using the steroid before arthroplasty [17,18].

## Material and methods

### Search strategy

To assemble all of the relevant published studies, PRISMA compliant searches of MEDLINE, EMBASE, ScienceDirect, OVID, the Cochrane CENTRAL database and Google scholar were performed for all peer-reviewed studies published through Aug 2014 that examine the relation between intraarticular steroid injections and infection rates in subsequent joint arthroplasty. The following search terms were used to maximize the search specificity and sensitivity: injection, arthroplasty, infection, replacement, steroid, hip and knee.

Secondary searches of the unpublished literature were conducted by searching the WHO International Clinical Trials Registry Platform, UK National Research Register Archive, and Current Controlled Trials from their inception to August 1, 2014. The reference lists of all the full text papers were examined to identify any initially omitted studies. We made no restrictions on the publication language.

### Inclusive and exclusive criteria

Studies were included if they compared the infection rates of joint arthroplasties in cohorts of joint arthroplasties that had previous intraarticular steroid injection, with the infection rates in cohorts of joint arthroplasties that had no previous steroid injection. Single case reports, reviews, and non-comparable studies were excluded.

### Study selection

Two reviewers (D.X. and Y. Y.) independently screened the titles and abstracts for the eligibility criteria. Subsequently, the full text of the studies that potentially met the inclusion criteria were read and the literature was reviewed to determine the final inclusion. We resolved disagreements by reaching a consensus through discussion.

### Date extraction

Two of the authors (D.X. and Y. Y.) independently extracted the following data from each full-text report using a standard data extraction form. The data extracted from studies included the title, authors, study design, prosthesis, steroid injection, time from injection to surgery, duration of follow-up, and outcomes parameters. The corresponding authors of the included studies were contacted to obtain any required information that was missing. The extracted data were verified by XL. M.

### Outcomes

Deep infection rate and superficial infection rate were the outcomes of the present study.

### Assessment of methodological quality

Following the Cochrane Handbook for Systematic Reviews of Interventions 5.0, the methodological quality of the included studies was independently assessed by two authors (D.X. and Y. Y.). Any disagreements were resolved by discussion. A third author (XL. M.) was the adjudicator when no consensus could be achieved. The methodological quality was assessed using the methodological index for non-randomized studies (MINORS) form [19], which was a valid instrument designed to assess the quality of comparative or non-comparative non-randomized controlled trials.

### Data analysis

We performed all of the meta-analyses with STATA 12.0 (Statacorp, college station, Tex). Odds ratio (OR) and 95% confidence intervals (CIs) were used to evaluate the dichotomous outcomes. A P-value <0.05 was considered statistically significant.

Statistical heterogeneity was assessed using Q statistics. A fixed-effects (inverse variance) model was used when the effects were assumed to be homogenous (*P* >0.05). *P* <0.05 implied statistical heterogeneity, and a random effects model was used in those circumstances.

### Evidence synthesis

The evidence grade was determined using the guidelines of the GRADE (Grading of Recommendations, Assessment, Development, and Evaluation) working group [17]. Although the GRADE system acknowledges the primacy of RCTs, it also recognizes circumstances in which observational studies generate high quality evidence of treatment effects [20]. The GRADE system uses a sequential assessment of the evidence quality that is followed by an assessment of the risk-benefit balance and a subsequent judgment on the strength of the recommendations. The evidence grades are divided into the following categories: (1) high, which indicates that further research is unlikely to alter confidence in the effect estimate; (2) moderate, which indicates that further research is likely to significantly alter confidence in the effect estimate and may change the estimate; (3) low, which indicates that further research is likely to significantly alter confidence in the effect estimate and to change the estimate; and (4) very low, which indicates that any effect estimate is uncertain. Uniformity of the estimated effects across studies and the extent to which the patients, interventions, and outcome measures are similar to those of interest may lower or raise the evidence grade. As recommended by the GRADE working group, the lowest evidence quality for any of the outcomes was used to rate the overall evidence quality. The evidence quality was graded using the GRADEpro Version 3.6 software. The strengths of the recommendations were based on the quality of the evidence.

## Results

### Search results

**Figure 1:**
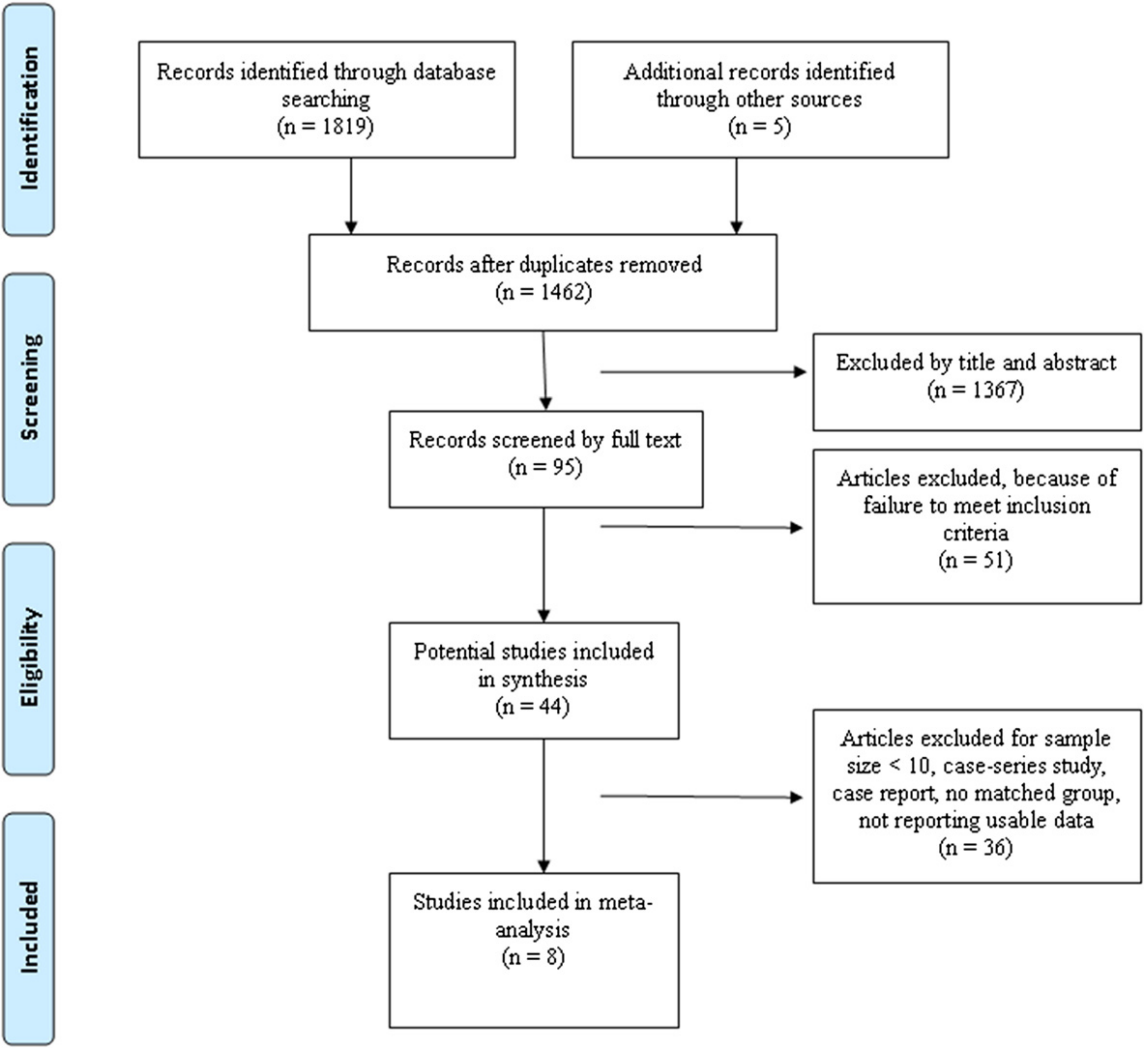
**The study selection and inclusion process.**

A total of 1461 titles and abstracts were preliminarily reviewed, of which 8 studies [13,14,16,21-25] eventually satisfied the eligibility criteria (Figure 1). One of the eight studies [22] published in abstract form. Six studies [13,14,16,22,23,25] evaluated infection rates in total hip arthroplasties (THA) and two studies [21,24] in total knee arthroplasties (TKA).

### Quality assessment

All studies had a high risk of bias resulting from study design limitations. The methodological quality assessments of the included studies used a MINORS form. The MINORS quality scores are presented in Table 1. The mean score was 11.8 (range, 8–14), which corresponded to a 49% score. This result indicated that there was considerable variability in the evidence base.

**Table 1:** **The study designs and MINORS appraisal scores for the included studies**

**Study**	**Study design**	**MINORS methodological criteria**												**Total**
		**1**	**2**	**3**	**4**	**5**	**6**	**7**	**8**	**9**	**10**	**11**	**12**	
Kaspar et al.	Retrospective cohort	2	0	0	1	1	2	0	0	2	2	2	1	13
Papavasiliou et al.	Retrospective cohort	2	1	0	1	0	2	1	0	2	2	1	1	13
McIntosh et al.	Retrospective cohort	2	0	0	1	1	2	0	0	2	2	1	1	12
Sreekumar et al.	Retrospective cohort	1	1	0	1	0	2	0	0	2	2	0	1	10
Desai et al.	Retrospective cohort	2	1	0	1	0	2	0	0	2	2	0	1	11
Haughton et al.	Retrospective cohort	2	1	0	2	0	1	1	0	2	2	1	1	13
Meermans et al.	Retrospective cohort	2	0	0	1	0	1	0	0	2	1	0	1	8
Croft et al.	Retrospective cohort	2	1	0	2	0	2	0	0	2	2	2	1	14

The MINORS criteria include the following items: (1) a clearly stated aim; (2) inclusion of consecutive patients; (3) prospective data collection; (4) endpoints appropriate to the aim of the study; (5) unbiased assessment of the study endpoint; (6) a follow-up period appropriate to the aims of the study; (7) less than 5% loss to follow-up; (8) prospective calculation of the sample size; (9) an adequate control group; (10) contemporary groups; (11) baseline equivalence of groups; and (12) adequate statistical analyses.

The items are scored as follows: 0 (not reported); 1 (reported but inadequate); or 2 (reported and adequate). The ideal global score for comparative studies is 24.

### Demographic characteristics

In total, eight retrospective cohort studies with 2909 total patients were eligible for inclusion. The individual sample sizes ranged from 80 to 1317 patients. All included studies reported the rate of deep infection after arthroplasty, while six also reported the rate of supercritical infection. The demographic characteristics of the included studies are summarized in Table 2.

**Table 2:** **The demographic characteristics of the included studies**

**Study**	**Study design**	**Prosthesis**	**Steroid injection**	**Time from injection to surgery**	**Deep infection rate**		**Superficial infection rate**		**Follow-up period**
					**Injected**	**Control**	**Injected**	**Control**	
Kaspar et al.	Retrospective cohort	THA	80 mg methylprednisolone	0.5-42.9 months	4/40	0/40	NR	NR	(9.9-86.2) months
Papavasiliou et al.	Retrospective cohort	TKA	NR	NR	3/54	0/90	12/54	10/90	1 year
McIntosh et al.	Retrospective cohort	THA	6–40 mg drug (not mentioned)	112 ± 81 days	3/224	1/224	11/224	8/224	NR
Sreekumar et al.	Retrospective cohort	THA	80 mg depomedrone	14 months	0/68	1/136	0/68	1/136	25-33 months
Desai et al.	Retrospective cohort	TKA	80 mg depomedrone	12 months	0/90	0/180	2/90	5/180	1-6 years
Haughton et al.	Retrospective cohort	THA	80 mg depomedrone or 40 mg triamcinolone	NR	4/254	14/1063	11/254	4/1063	NR
Meermans et al.	Retrospective cohort	THA	80 mg depomedrone +5–15 mg levobupivacaine	12 months	1/175	1/175	5/175	7/175	12-131 months
Croft et al.	Retrospective cohort	THA	40 mg depomedrol +4 ml 2% xylocaine	5.9 months	0/48	0/48	NR	NR	1.4-54.1 months

THA, total hip arthroplasty; TKA, total knee arthroplasty; NR, not reported.

### Outcome analyses

#### Deep infection rate

**Figure 2:**
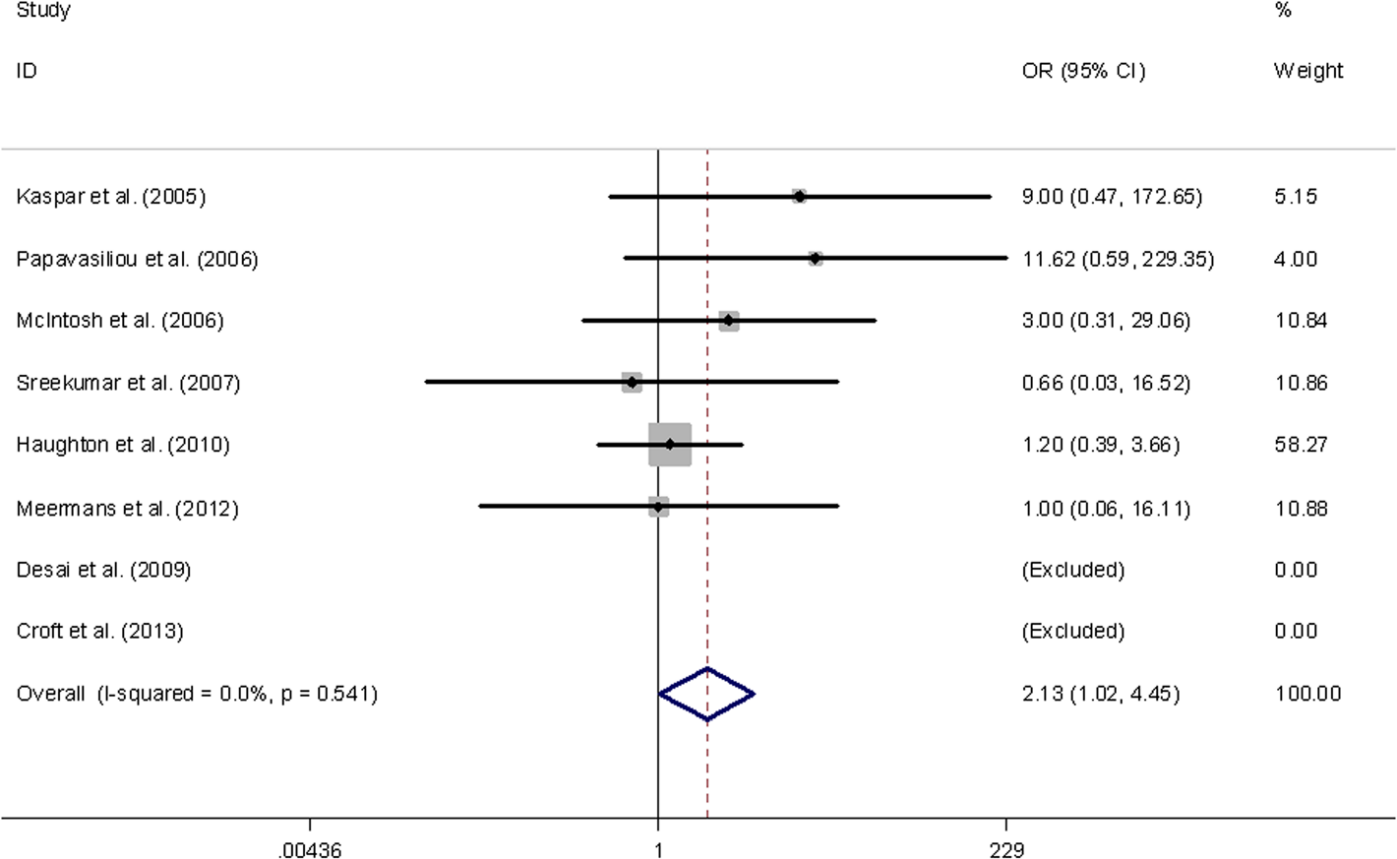
**The odds ratio (OR) estimate for deep infection rate.**

The deep infection rates for each analyzed studies were 0–10% in the injected group and 0–1.3% in the control group respectively. Meta-analysis showed that patients with steroid injection before arthroplasty had a higher deep infection rate than patients without steroid injection (OR = 2.13, 95% CI 1.02-4.45) (Figure 2).

#### Superficial infection rate

**Figure 3:**
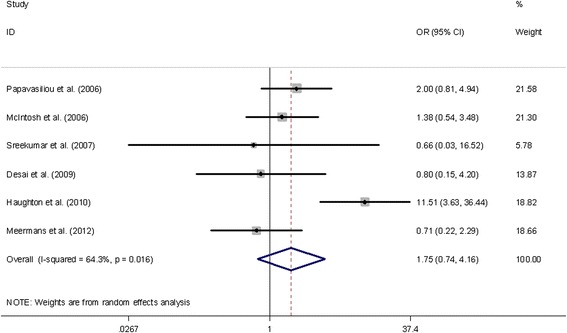
**The odds ratio (OR) estimate for superficial infection rate.**

The superficial infection rates for each analyzed studies were 0–22.2% in the injected group and 0.4–11.1% in the control group respectively. Meta-analysis showed that steroid injection prior to joint arthroplasty had no significant effect on superficial infection rate (OR = 1.75, 95% CI 0.74-4.16) (Figure 3).

### Quality of the evidence and recommendation strengths

The two outcomes in this meta-analysis were evaluated using the GRADE system. The evidence quality for each outcome was low or very low (Table 3). Therefore, we agreed that the overall evidence quality was very low. This finding may lower the confidence in any recommendations.

**Table 3:** **The GRADE evidence quality for each outcome**

**Quality assessment**							**No. of patients**		**Effect**	**Quality**	**Importance**
**Outcomes**	**No of studies**	**Risk of bias**	**Inconsistency**	**Indirectness**	**Imprecision**	**Other considerations**	**Injected**	**Control**	**OR (95% CI)**		
Superficial infection rate	6	Serious	Serious	None serious	None serious	None	41/865(4.7%)	35/1868(1.9%)	OR 1.75 (0.74-4.16)	⊕○○○ VERY LOW	Important
Deep infection rate	8	None serious	None serious	None serious	None serious	None	15/953 (1.6%)	17/1956 (0.9%)	OR 2.13 (1.02-4.45)	⊕ ⊕ ○○ LOW	Important

OR, odds ratio.

## Discussion

Infection is a major complication of a total joint arthroplasty. It occurs in only a small number of patients but results in substantial morbidity and a decline in functional outcome. An increased risk of postoperative infection after intraarticular steroid injection has been previously questioned with some clinical studies [13,14]. Several studies reported that intraarticular steroid injection may lead to increase arthroplasty infection rates. This may be due to failure of the steroid to dissolve which may thus persist and cause local immunosupression following joint arthroplasty [13,24]. Several studies reported that it may be related to contamination of the joint by the injection process [26]. However, Other studies demonstrated that there was no association between previous steroid injections and infection after subsequent joint arthroplasty [16,21,23]. Because infection rate of joint arthroplasty is an infrequent event [27], the failure of some studies that demonstrate such association may be related to the small number of patients included and hence low statistical power.

Meta-analysis is used as the main method in the research paper. It is more accurate and reliable than regression analysis or original papers. Meta-analysis can enhance statistical power and enlarger sample size by combining original studies, which could provide more robust evidence. Therefore, we conducted a meta-analysis to evaluate the evidence from relevant studies that examine the relation between intraarticular steroid injections and infection rates in subsequent joint arthroplasty. Furthermore, there have been no guidelines or recommendations for intraarticular steroid injections before joint arthroplasty. Therefore, there is a need for an evidence base to help surgeons make clinical decisions and develop optimal treatments before joint arthroplasty. To the best of our knowledge, this study is the first meta-analysis to use the GRADE system to evaluate the quality of the evidence evaluating the influence of intraarticular steroid injections on infection rate before joint arthroplasty.

Because of the challenges clinicians face from the lack of randomized surgical trials and the large number of observational surgical studies, retrospective studies were included in this meta-analysis. However, including retrospective studies in the present study introduces a high risk of bias. The methodological quality assessment identified a number of limitations to the current evidence base. Combining the results of the observational studies could cause significant bias. To some extent, the observational studies included in this study may overestimate the actual effect. Moreover, confounding factors that should be balanced by randomized methods disturbed the intervention effect in the retrospective studies. Therefore, most of the included studies had relatively high methodological assessment risks, which may have influenced the accuracy and reliability of the pooled results.

Some degree of clinical heterogeneity was induced by the different surgical technologies used, surgical site, medical co-morbidities, nutritional status of patients, surgical duration, admission to the hospital from a healthcare facility, pre-surgical medical status, follow-up times, surgeon’s experience, use of drains and diagnostic criteria for indection. Heterogeneity may have been caused by poor study design. Because of limited information got from original studies, heterogeneity cannot be completely resolved. Accordingly, although the results of the meta-analysis should be considered appropriate, methodological quality defects and clinical heterogeneity should be considered when interpreting the findings.

The most important finding of the present meta-anslysis was that intraarticular steroid injection had statistically significant effect on the deep infection rates of subsequent joint arthroplasty but not on superficial infection rates, suggesting that such practice should not be taken lightly. Papavasiliou et al. [24] showed a statistically higher deep infection rate in TKA that had a previous steroid injection as compared to those who had no previous injection. However, Desai et al. [21] showed no increased incidence of deep or superficial infection in TKA after a prior steroid injection. Meermans et al. found no differences between the injected and control groups for the rate of deep or superficial infection in THA.

Steroids can provide symptomatic control in the short term and may allow surgery to be delayed until a more appropriate time. The problem is that it is not possible to predict which patients will respond positively to an injection. Several studies [13,14] found a greater rate of infection in THA performed after a previous steroid injection. However, it is unclear which component of the injection procedure may be culpable: the arthrography dye, the steroid or its depot vehicle, contamination of the local anesthetic, the invasiveness of a needle through prepared skin, any breech of sterile technique, or the time between the steroid injection and the arthroplasty [23]. Because of poor study design and limitations, the GRADE evidence quality for deep and superficial infection rate was low and very low respectively. Because of the insufficient quality of evidence, the effect estimate is uncertain and has a lower GRADE recommendation strength.

In the present study, the GARDE evidence quality was very low, which means that we were very uncertain about the estimates. The lowest GRADE evidence quality will lower our confidence in recommendation. Therefore, we would better make clinical decision based on individual characteristics of the patients.

The primary limitations of this meta-analysis include the following: (1) the statistical efficacy could be improved by including more studies. Owing to the finite of included studies, subgroup analysis can not be performed on TKA or THA. It may exert instability on consistency of outcomes. (2) poorly designed retrospective studies were more likely to suffer from various types of bias. The pooled results may have significant bias originated from limitations of original retrospective studies; (3) To some extent, clinical heterogeneity can not be resolved completely, such as injection protocols, timing of injections, number of injections and surgical experience. (4) the overall GRADE quality of evidence was very low, which lowers confidence in any subsequent recommendations. Although we used the GRADE system to evaluate the evidence quality and recommendation strengths, judgment is still required.

## Conclusion

From this meta-analysis and grading of the evidence, the present study offers useful conclusions and demonstrates that intraarticular steroid injections may lead to increased deep infection rates of subsequent joint arthroplasty but not the superficial infection rates. However, the overall GRADE evidence quality was very low, which will lower our confidence in recommendation strengths. Further high-quality studies are still required to validate the results.
